# CTGF Promotes the Osteoblast Differentiation of Human Periodontal Ligament Stem Cells by Positively Regulating BMP2/Smad Signal Transduction

**DOI:** 10.1155/2022/2938015

**Published:** 2022-09-15

**Authors:** Shuyun Yan, Meng Zhang, Guimei Yang, Yumei Sun, Dongmei Ai

**Affiliations:** Department of Stomatology, The Second Affiliated Hospital of Shandong University of Traditional Chinese Medicine, China

## Abstract

**Objective:**

This work is aimed at revealing the role and the molecular mechanism of connective tissue growth factor 2 (CTGF) in the osteoblast differentiation of periodontal ligament stem cells (PDLSCs).

**Methods:**

The osteogenic differentiation of PDLSCs was induced by osteogenic induction medium (OM), and the expression level of osteogenic related proteins ALP, RUNX2, OCN, and CTGF was estimated using quantitative real-time polymerase chain reaction (qRT-PCR) and Western blotting analysis. We constructed cell lines with CTGF overexpression or knockdown to verify the role of CTGF in the osteoblast differentiation of PDLSCs. Alkaline phosphatase (ALP) staining was introduced to measure the osteoblasts activity, and alizarin red S (ARS) staining was employed to test matrix mineralization. The interaction between CTGF and bone morphogenetic protein-2 (BMP-2) was determined by endogenous coimmunoprecipitation (Co-IP).

**Results:**

The expression level of CTGF was increased during the osteogenic induction of PDLSCs. Additionally, CTGF overexpression effectively maintained the stemness and facilitated the osteoblast differentiation in PDLSCs, and CTGF knockdown exerted opposite effects. Moreover, at molecular mechanism, CTGF increased the activity of BMP-2/Smad signaling pathway.

**Conclusion:**

This investigation verified that CTGF promotes the osteoblast differentiation in PDLSCs at least partly by activating BMP-2/Smad cascade signal.

## 1. Introduction

Periodontitis, a chronic and infectious disease, is mainly caused by the loss of alveolar bone and aberrant vascularization [1]. Previous studies have illustrated that severe periodontitis already affects 9 to 11 percent of adults worldwide [2]. Conventional treatment methods such as root planning and induced bone regeneration have not achieved satisfactory clinical results [3]. The periodontal ligament could be a soft tissue in the alveolar fossa that improves tooth nutrition and promotes alveolar bone remodeling [4, 5]. Periodontal ligament stem cells (PDLSCs) rooted from periodontal ligament tissue are identified as a group of mesenchymal stem cells (MSCs) and have the potential for self-renewal and multidirectional differentiation [6]. Current studies pointed that PDLSCs exhibit different differentiation abilities and serve as the most crucial candidate stem cells for inducing periodontal regeneration [7–9]. It has been reported that PDLSCs ameliorate periodontal intra bone loss. Besides, osteogenic differentiation of PDLSCs is crucial for repairing alveolar bone loss as well as tooth defects [10–13]. Increasing evidence indicates that various factors including mechanical vibration, electromagnetic field, and genes are able to affect the process of osteogenic differentiation of PDLSCs [14–17]. Therefore, exploring novel targets to regulate PDLSCs osteogenic differentiation is an important goal for the treatment of periodontitis.

It is reported that multiple signaling pathways such as mitogen-activated protein kinase (MAPK) pathway [18] and Wnt/*β*-catenin pathway regulate the osteogenic differentiation progression [19]. Bone morphogenetic proteins (BMPs), the essential components of the transforming growth factor -*β* (TGF-*β*) superfamily, have been proved to be closely associated with the osteogenic differentiation of MSCs [20]. BMP-2 was first noted for its ability to induce ectopic bone and chondrogenesis [21]. Recent investigations demonstrated that BMP-2 effectively accelerate the osteoblast differentiation of PDLSCs [22]. Zhang's team revealed that BMP-2 increases the expression of osteogenic differentiation-related proteins RUNX2, ALP, Colla, and Oc, thereby facilitating bone formation [23]. Mechanistically, activated BMP-2 phosphorylates Smad pathway such as Smad1/5/8 and ultimately activates the expression of downstream target genes including RUNX2 [24, 25]. However, how BMP-2/Smad pathway is regulated in the osteoblast differentiation of PDLSCs needs to be further explored.

Connective tissue growth factor 2 (CTGF, CCN2), a member of the CCN family, is closely related to a variety of biological processes, including angiogenesis and wound healing [26, 27]. Increasing evidence revealed that CTGF accelerates the production of extracellular matrix components and stimulates the differentiation of chondrogenic MSC [28–30]. Wang et al. verified that CTGF overexpression facilitates the osteogenic differentiation of MSCs [31]. Lee et al. revealed that CTGF accelerates the differentiation of human MSCs into fibroblasts and influences connective tissue healing in rodents [32]. Asano et al. discovered that CTGF possess the ability to promote the growth and differentiation of mouse PDL cells [33]. Additionally, Yuda et al. proved that CTGF accelerates the proliferation, cycle, migration, and osteoblast differentiation of a clonal cell line (1-11) by constructing and isolating fibroblast cell lines [34]. However, the effect of CTGF on the differentiation of PDLSCs into osteoblasts remains uncovered. Moreover, increasing findings have indicated that CTGF is able to negatively regulate BMP-2 in mouse osteoblasts but antagonizes and systemizes BMP-2 signaling in chondrocytes [35, 36]. Nevertheless, whether and how CTGF regulates BMP-2/Smad pathway in the osteoblast differentiation of PDLSCs is still unclear.

This work revealed that CTGF facilitates the osteoblast differentiation of PDLSCs at least partly by upregulating BMP-2/Smad cascade signaling.

## 2. Materials

### 2.1. Cell Culture and Induction

Primary human PDLSCs were obtained as introduced in previous reporters [20, 37]. Premolars extracted from healthy people after orthodontics are preserved. The periodontal ligament tissue was scraped from 1/3 of the root of the premolars and placed in a petri dish containing fresh *α*-minimum primary medium (*α*-MEM, P03-2610, PAN, Germany) in advance. Next, a scalpel was used to separate 1 mm^3^ of periodontal tissues, and the tissues were cultured in a culture flask supplemented with complete *α*-MEM (*α*-MEM, P03-2610, PAN, Germany) containing 10% fetal bovine serum (FBS, DX101, DENING BIO, China). Place the flask upside down in a CO_2_ incubator (S@feGrow 188, BioMatrix Life Science (Qingdao) Co., Ltd., China) at 37° C. After 4 hours, the tissues in the flask were turned over once and then were serial subcultured. Then, PDLSCs were purified using the monoclonal screening approach. After 3-5 generations, PDLSCs were frozen and stored, or further studies were carried out. After the cells reached 70-80% density, the original growth medium (*α*-MEM, also known as GM) was replaced with osteogenic induction medium (OM), *α*-MEM medium containing 100 nM dexamethasone (HY-14648, MCE, USA), 200 *μ*M L-ascorbic acid (HY-B0166, MCE, USA), and 2 mM *β*-glycerophosphate (G9422, Sigma-Aldrich, USA). The old OM was replaced every 2 days. All patients have signed informed consent, and all protocols were permitted by the Ethic Committee of The Second Affiliated Hospital of Shandong University of Chinese Medicine.

### 2.2. Cell Transfection

The transfected small interfering RNAs (siRNAs) were obtained from Zoonbio Biotechnology (China), and plasmids were obtained from Kingsley Biotechnology Co., Ltd. (China). When the cell density reached 50-60%, PDLSCs were transfected with specific vectors employing Lipofectamine 2000 Transfection Reagent (11668030, Invitrogen, USA) following the instruction of the manufacturer. CTGF overexpression lentivirus and CTGF siRNA lentivirus were obtained by GeneChem (China) and transduced into PDLSCs following the instructions. The sequences of siRNAs are listed in Table 1.

### 2.3. Measurement of the Activity of Antioxidative Stress Markers

PDLSCs transfected with indicated vectors were divided into 3 groups: control group, lipopolysaccharide (LPS, 10 *μ*M, L8880, Solarbio, China) group, and LPS + pcDNA3.1/CTGF group or LPS + si-CTGF-1# group. After overexpression of 24 h, the content of superoxide dismutase (SOD) or glutathione (GSH) was estimated utilizing superoxide dismutase activity assay kit (BC5165, Solarbio, China), microreduced glutathione (GSH) assay kit (BC1175, Solarbio, China), or malondialdehyde (MDA) content detection kit (BC0020, Solarbio, China) following the kit's instruction, respectively.

### 2.4. QRT-PCR

The total RNA from PDLSCs was collected at 0, 7, and 14 days after osteogenic induction using TriQuick Reagent (R1100, Solarbio, China) abiding by the kit's protocol. Next, 1 *μ*g total RNA was employed to synthesize complementary DNA (cDNA), and then, the cDNA was used to amplify the targeted gene utilizing a BeyoFast™ SYBR Green One-Step qRT-PCR Kit (D7268S, Beyotime, China) according to the manufacturer's instruction. The relative expression of each RNA was estimated via 2^–ΔΔCT^ method, and *β*-actin was considered as the control gene. The sequences of primers are presented in Table 2.

### 2.5. Western Blotting

PDLSCs transfected with indicated vectors were harvested utilizing RIPA Lysis Buffer (P0013C, Beyotime, China) following the instruction of the regent. Then, the lysate was centrifuged at 4°C (12000 rpm, 10 min), and total protein was isolated with 12% SDS-PAGE. Next, the blots were shifted onto polyvinylidene fluoride (PVDF) membrane (YA1701-1EA, Solarbio, China), followed by blocking with 5% nonfat powdered milk (D8340, Solarbio, China) dissolving in TBS with Tween-20 (ST671, Beyotime, China) solution for 40 min. The membranes were treated with antibody anti-CTGF antibody (ab6992, Abcam, UK), ANTI-FLAG(R) antibody produced in rabbit (F7425, MilliporeSigma, USA), anti-Nanog antibody (ab80892, Abcam, UK), anti-SOX2 antibody (ab97959, Abcam, UK), c-Myc Rabbit mAb (A19032, Abclonal, China), anti-RUNX2 antibody (ab23981, Abcam, UK), anti-collagen I antibody (ab34710, Abcam, UK), anti-ALP antibody (ab83259, Abcam, UK), anti-BMP2 antibody (ab14933, Abcam, UK), Phospho-Smad1 (Ser463/465)/Smad5 (Ser463/465)/Smad9 (Ser465/467) (D5B10) Rabbit mAb (13820, Cell signaling Technology, USA), or anti-beta actin antibody (ab8227, Abcam, UK) overnight at 4°C. Finally, HRP-labeled Goat anti-Rabbit IgG(H + L) (A0208, Beyotime, China) and BeyoECL Plus (P0018S, Beyotime, China) were employed to observe the protein bands.

### 2.6. Alkaline Phosphatase (ALP) Staining

ALP staining analysis was carried out employing an alkaline phosphatase (ALP/AKP) test kit (ml092964, Shanghai Enzyme Linked Biotechnology Co., Ltd.) after 7 days of osteogenic induction according to the manufacturer's instruction.

### 2.7. ALP Activity

The ALP activity was determined as described in the previous study [37]. In brief, PDLSCs transfected with indicated vectors were washed with precooled phosphate buffer saline (PBS, C0221A, Beyotime, China); then, the cells were permeated with 1% Triton X-100 (ST797, Beyotime, China) and scraped into distilled water using a sterilized cell scraper. Next, a kit (P0012S, Beyotime, China) was used to estimate the protein concentration, and the activity of ALP was calculated according to the absorbance at 405 nm.

### 2.8. Alizarin Red S (ARS) Staining

ARS staining is utilized to determine the formation of mineralized nodules using an osteoblast-mineralized nodule staining kit (C0148S, Beyotime, China) according to the kit's protocol. Briefly, PDLSCs transfected with indicated vectors were immobilized with stationary liquid for 30 min at day 14 of osteogenic induction. Then, the cells were stained by utilizing the ARS solution for another 20 min. Next, the ARS staining solution was dissolved in 10% cetylpyridine chloride (C9002, Sigma-Aldrich, USA) for 1 hour, and the absorbance of the solution at 570 nm was measured to determine the degree of mineralized nodules.

### 2.9. Coimmunoprecipitation (Co-IP) Assay

PDLSCs were collected using the cell lysis buffer for Western and IP (P0013, Beyotime, China). The lysates were incubated with protein A + G agarose (P2012, Beyotime, China) combined with Rabbit Control IgG (AC005, Abclonal, China), anti-CTGF antibody (ab6992, Abcam, UK), or anti-BMP2 antibody (ab14933, Abcam, UK) overnight at 4°C. After washing with lysis buffer for 3 times, the solution was centrifuged at 4°C for 5 min (12000 rpm), and the supernatant was removed. Then, the agarose with IgG, CTGF, or BMP-2 was lysed utilizing SDS lysis buffer (P0013G, Beyotime, China), and the proteins were isolated as described in Western blotting section.

### 2.10. Statistical Analysis

The statistical analysis was conducted by comparing mean ± standard deviation (SD) employing a two-tailed Student's *t* test (two groups) or one-way analysis of variance (ANOVA) combined with Tukey's test (multiple groups). Additionally, the significance was indicated with ^∗∗∗∗^*p* < 0.0001, ^∗∗∗^*p* < 0.001, and ^∗∗^*p* < 0.01.

## 3. Results

### 3.1. CTGF Is Highly Expressed during the Osteoblast Differentiation of PDLSCs

**Figure 1:**
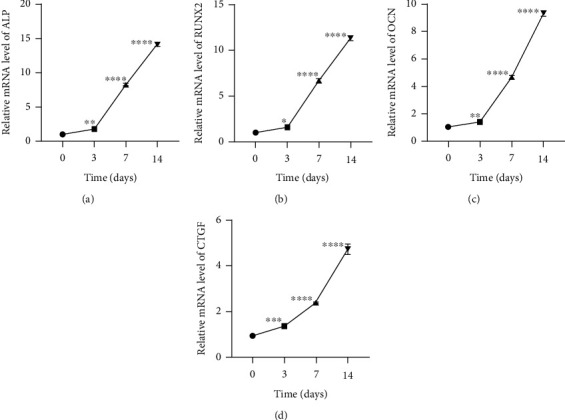
CTGF is highly expressed during the osteoblast differentiation of PDLSCs. (a–c) The content of ALP, RUNX2, and OCN detected via qRT-PCR (^∗∗∗∗^*p* < 0.0001, ^∗∗^*p* < 0.01). (d) The content of CTGF detected via qRT-PCR (^∗∗∗∗^*p* < 0.0001, ^∗∗∗^*p* < 0.001). ^∗∗∗∗^*p* < 0.0001, ^∗∗∗^*p* < 0.001, and ^∗∗^*p* < 0.01 versus 0 day.

PDLSCs were maintained in osteogenic medium for 14 days, and the osteogenic induction efficiency was verified by qRT-PCR analysis. The data illustrated the expression level of osteogenic related proteins ALP, RUNX2, and OCN was obviously upregulated during the induction (Figures 1(a)–1(c)). In addition, the CTGF expression was increased notably with the process of osteogenic induction (Figure 1(d)). Collectively, these data suggested that CTGF might be correlated to the osteogenic differentiation of PDLSCs.

### 3.2. CTGF Affects the Stemness of PDLSCs and Regulates Oxidative Stress

**Figure 2:**
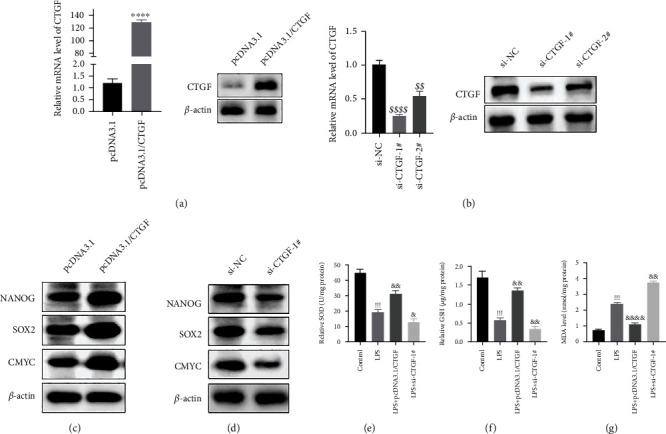
CTGF affects the stemness of PDLSCs. (a) The content of CTGF measured via Western blotting analysis (^∗∗∗∗^*p* < 0.0001). (B) The content of CTGF determined via Western blotting analysis (^$$$$^*p* < 0.0001, ^$$^*p* < 0.01). (c) The content of NANOG, SOX2, and CMYC detected via Western blotting analysis. (d) The content of NANOG, SOX2, and CMYC determined via Western blotting analysis (e, f) The production of SOD (e), GSH (f), and MDA (g) estimated via specific kit (^!!!!^*p* < 0.0001, ^!!!^*p* < 0.001, ^&&&&^*p* < 0.0001, ^&&^*p* < 0.01, ^&^*p* < 0.05). ^∗∗∗∗^*p* < 0.0001, ^∗∗∗^*p* < 0.001 versus pcDNA3.1. ^$$$$^*p* < 0.0001, ^$$^*p* < 0.01 versus si-NC. ^!!!!^*p* < 0.0001, ^!!!^*p* < 0.001 versus control. ^&&&&^*p* < 0.0001, ^&&^*p* < 0.01, ^&^*p* < 0.05 versus LPS.

To explore the role of CTGF in PDLSCs, we constructed PDLSC lines with overexpression (Figure 2(a)) or knockdown (Figure 2(b)) of CTGF. Besides, as exhibited in Figure 2(b), si-CTGF-1# presented the highest efficiency and was chosen to conduct subsequent experiments (Figure 2(b)). Firstly, we determined the effects of CTGF on the stemness of PDLSCs by measuring the expression level of stemness associated genes. Interestingly, overexpression of CTGF dramatically upregulated the content of NANOG, SOX2, and CMYC in PDLSCs (Figure 2(c)). Consistently, CTGF silence notably downregulated the load of these genes (Figure 2(d)). Subsequently, we tested the roles of CTGF in oxidative stress in PDLSCs. With LPS treatment, the production of oxidative stress-associated markers SOD as well as GSH was dramatically decreased, while the level of MDA was increased notably in PDLSCs. Besides, overexpression of CTGF effectively reversed these effects on PDLASCs. S. However, CTGF silence further reduced the secretion of SOD and GSH (Figures 2(e)–2(g)). These findings indicated that CTGF effectively maintains the stemness of PDLSCs and might protect PDLSCs from oxidative stress to some extent.

### 3.3. CTGF Overexpression Promotes the Osteoblast Differentiation of PDLSCs

**Figure 3:**
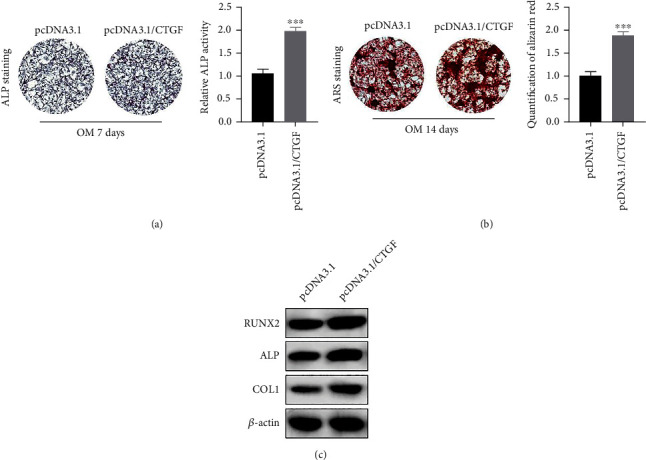
CTGF overexpression promotes the osteoblast differentiation of PDLSCs. (a) ALP staining and ALP activity (^∗∗∗^*p* < 0.001). (b) Images of ARS staining and quantification of ARS (^∗∗∗^*p* < 0.001). (c) The content of RUNX2, ALP, and COL1 detected via Western blotting analysis. ^∗∗∗^*p* < 0.001 versus pcDNA3.1.

To investigate the influence of CTGF on the osteogenic ability of PDLSCs, osteogenesis was induced again, and ALP staining analysis was performed. The data demonstrated that CTGF overexpression dramatically enhanced the ALP staining and ALP activity after osteogenic induction for 7 days (Figure 3(a)). After 14 days of osteogenic induction, ARS staining assay showed that overexpression of CTGF increased the amount of ARS staining remarkably, which implied that CTGF overexpression enhanced the matrix mineralization in PDLSCs (Figure 3(b)). Additionally, the expression level of proteins related to osteoblast differentiation was tested. The data illustrated that CTGF obviously increased the expression of RUNX2, ALP, and COL1 in PDLSCs (Figure 3(c)). These findings demonstrated that overexpression of CTGF facilitates the osteoblast differentiation in PDLSCs.

### 3.4. CTGF Knockdown Inhibits the Osteoblast Differentiation of PDLSCs

**Figure 4:**
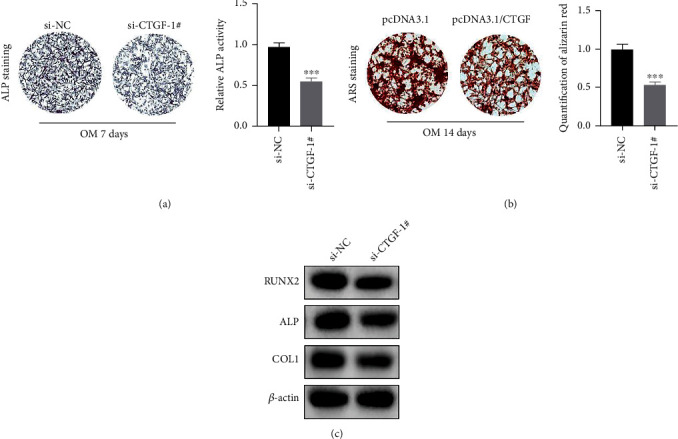
CTGF knockdown inhibits the osteoblast differentiation of PDLSCs. (a) Images of ALP staining and the activity of ALP (^∗∗∗^*p* < 0.001). (b) Images of ARS staining and quantification of ARS (^∗∗∗^*p* < 0.001). (c) The expression level of RUNX2, ALP, and COL1 detected via Western blotting analysis. ^∗∗∗^*p* < 0.001 versus si-NC.

To further prove the role of CTGF in PDLSC osteogenic differentiation, we conducted above-mentioned experiments in PDLSCs with CTGF silence. As expected, CTFG knockdown dramatically reduced the intensity of ALP staining and ALP activity at 7 days after osteogenic induction (Figure 4(a)). After 14 days, decrease of matrix mineralization was observed in the CTGF-knockdown group by performing ARS staining analysis (Figure 4(b)). Additionally, Western blotting analysis revealed that the protein level of RUNX2, ALP, and COL1 was downregulated via CTGF silence obviously. Overall, these results confirmed that knockdown of CTGF suppressed the osteogenic differentiation of PDLSCs.

### 3.5. CTGF Promotes the Osteoblast Differentiation in PDLSCs via the BMP-2/Smad Pathway

**Figure 5:**
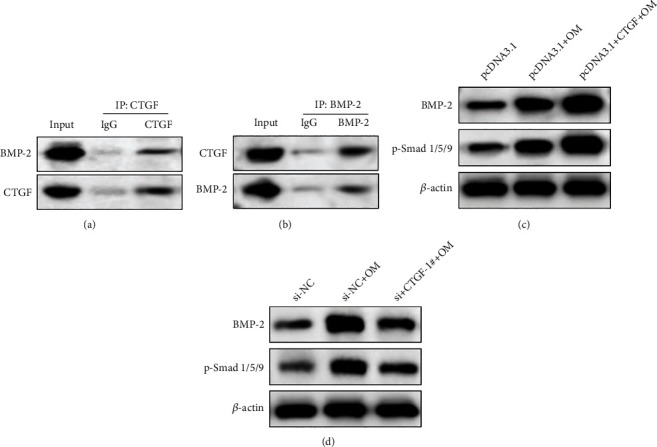
CTGF promotes the osteoblast differentiation in PDLSCs via the BMP-2/Smad pathway. (a, b) The interaction between CTGF and BMP-2 detected via Co-IP analysis. (c) The content of BMP-2 as well as p-Smad1/5/9 detected via Western blotting analysis. (d) The content level of BMP-2 as well as p-Smad1/5/9 detected via Western blotting analysis.

To verify whether CTGF affects the osteogenic differentiation of PDLSCs by mediating BMP-2/Smad signal, we firstly performed endogenous Co-IP analysis to determine the relationship between CTGF and BMP-2 in PDLSCs. As presented in Figures 5(a) and 5(b), endogenous BMP-2 in PDLSCs was immunoprecipitated by CTGF antibody. Besides, the direct interaction was further proved via immunoprecipitation of CTGF with BMP-2 antibody (Figures 5(a) and 5(b)). Moreover, CTGF overexpression notably increased the BMP-2 expression and the phosphorylation of p-Smad1/5/9 after osteogenic induction (Figure 5(c)). In addition, CTGF knockdown reduced the content of BMP-2 and p-Smad1/5/9 after osteogenic induction remarkably (Figure 5(d)). Taken together, these findings revealed that CTGF facilitates the osteoblast differentiation of PDLSCs at least partly by promoting BMP-2/Smad cascade signal.

## 4. Discussion

In summary, this investigation revealed that the CTGF expression was increased during osteogenic induction. Additionally, CTGF overexpression enhanced the stemness of PDLSCs, while CTGF silence impaired that notably. Moreover, overexpression of CTGF facilitated the osteoblast differentiation in PDLSCs, whereas knockdown of CTGF exerted opposite effects obviously. Furthermore, CTGF effectively upregulated the activities of BMP-2/Smad pathway by interacting with BMP-2 directly.

Recently, the application of MSCs plays a crucial role in the field of regenerative medicine. Besides, bone marrow MSCs and adipogenic stem cells are regarded as two types of MSCs that are widely investigated in clinical practice [38]. It is of great clinical significance to further explore the underlying mechanism of ASCs. In addition, PDLSC, as a superior regenerative cell in periodontal tissue, has been reported to have multidifferentiation abilities [6, 39]. It is identified as an ideal cell type for exploring periodontal tissue as well as bone regeneration [40]. Thus, it is vital to elucidate the molecular mechanism of its multivalent differentiation.

It has been reported that CTGF is tightly associated with the repair of a variety of tissues including bone regeneration [41, 42]. Besides, previous studies have reported that CTGF is able to promote the differentiation of bone marrow MSCs into tendon fibroblasts [43]. Asano et al. discovered that CTGF effectively accelerates the tooth development of mice and also promotes the progression of PDL cells [33, 44]. Recent evidence suggests that the expression of CTGF in human PDL cells will be dramatically increased under tensile loading [34]. Nevertheless, the role of CTGF in the osteogenic differentiation of PDLSCs has not been expounded now. In this work, we carried out osteoblast induction in PDLSCs and revealed that the expression of CTGF was upregulated obviously with the process of induction. Meanwhile, interestingly, overexpressed CTGF notably increased the content of stemness-associated proteins including NANOG, SOX2, and CMYC but CTGF knockdown reduced the expression of NANOG, SOX2, and CMYC in PDLSCs. These findings implied that CTGF could ensure PDLSCs more differentiational potentials.

Additionally, emerging evidence revealed that the occurrence of oxidative stress is closely associated with PDLSC osteoblast differentiation [45], and CTGF has been proved to participate in oxidative stress in various biological progresses [46]. However, the detailed role of CTGF in PDLSCs remains unclear. LPS, as a cell wall component of Gram-negative bacteria, is a vital factor contributing to oxidative stress and periodontitis [47]. Moreover, SOD, GSH, and MDA is verified the crucial biomarker of oxidative stress [48, 49]. SOD clears away superoxide anion radical and maintain the balance between oxidation and antioxidation [50]. GSH serves as a scavenger to reduce the content of O_2_ and H_2_O_2_. [51]. Besides, the accumulation of MDA also signifies the occurrence of oxidative stress [52]. Consistently, this work proved that LPS treatment decreased the production of SOD and GSH while increased MDA level in PDLSCs. Nevertheless, CTGF overexpression effectively reversed these effects of LPS, whereas CTGF knockdown further aggravated those in PDLSCs. These results indicated that CTGF exerted its role in PDLSCs might by inhibiting LPS-induced oxidative stress to some extent.

Subsequently, this work demonstrated that overexpressed CTGF notably increased the intensity of ALP and ARS staining, which is identified as the significant symbol of osteoblast differentiation [37]. Similar to previous investigation [53], the increased activity of ALP and upregulated expression of osteogenic proteins (RUNX2, COL1, and ALP) was observed after CTGF overexpression. Moreover, knockdown of CTGF exhibited opposite effects in the osteoblast differentiation of PDLSCs. These results preliminarily verified that CTGF promotes the osteoblast differentiation in PDLSCs.

It is well-known that BMPs exerted essential effects on the regulation of cascade signal associated with the progression of bone formation [25], and the role was modulated via Smad pathway [54]. Increasing evidence proved that CTGF regulates diverse biological progressions including cell viability and migration as well as differentiation by interacting with various growth factors or matrix proteins [55], which are vital for osteogenic differentiation. Previous investigation revealed that CTGF negatively regulated BMP-2 pathway in the osteoblast [35]. Maeda et al. found that CTGF exerted both antagonistic role and agonistic role for BMP-2 in chondrocyte [36]. Nevertheless, the effects of CTGF for BMP-2 in the osteogenic differentiation of PDLSCs remain unknown. In this study, we found that CTGF interacted with BMP-2 directly. Additionally, overexpression of CTGF increased the expression of BMP-2 and the phosphorylation of Smad1/5/9, while CTGF knockdown decreased the activity of BMP-2/Smad pathway effectively during the osteogenic induction in PDLSCs. The data suggested that CTGF dramatically promotes BMP-2/Smad signal in the osteoblast differentiation of PDLSCs.

However, there existed some limitations in our present study. For example, whether CTGF facilitates osteogenic differentiation *in vivo* needs to be further verified. Moreover, whether and how CTGF plays its inducible role in the osteoblast differentiation of PDLSCs by regulating oxidative stress needs to be further verified and investigated in the subsequent study. Furthermore, more experiments will be conducted to prove how CTGF regulates BMP-2/Smad signaling pathway in PDLSCs. Additionally, we mean to explore whether CTGF affect the other cascade signal mediated via BMP-2 in the subsequent study.

## 5. Conclusion

This investigation verified that CTGF facilitates the osteogenic differentiation of PDLSCs at least partly by activating BMP-2/Smad signaling pathway.

## Figures and Tables

**Table 1:** The sequences of siRNAs.

si-RNA	Forward (5′-3′)	Reverse (5′-3′)
si-NC	GTTCTCCGAACGTGTCACGT	ACGTGACACGTTCGGAGAAC
si-CTGF-1#	GGUCAAGCUGCCCGGGAAATT	UUUCCCGGGCAGCUUGACCTT
si-CTGF-2#	GCACCAGCAUGAAGACAUA	UAUGUCUUGAUGCUCCUGC
si-CTGF-3#	CCAGACCCAACUAUGAUUA	UAAUCAUAGUUGGGUVUGG

**Table 2:** The sequences of qRT-PCR primers.

Gene	Forward	Reverse
RUNX2	5′-ACTACCAGCCACCGAGACCA-3′	5′-ACTGCTTGCAGCCTTAAATGACTCT-3′
OCN	5′-ACCCTGACCCATCTCAGAAGCA-3′	5′-CTTGGAAGGGTCTGTGGGGCTA-3′
ALP	5′-GAACGTGGTCACCTCCATCCT-3′	5′-TCTCGTGGTCACAATGC-3′
CTGF	5′-GGCCTCTTCTGCGATTTCG-3′	5′-GCAGCTTGACCCTTCTCGG-3′
*β*-Actin	5′-GTGACGTTGACATCCGTAAAGA-3′	5′-GCCGGACTCATCGTACTCC-3′

## Data Availability

The data used to support the findings of this study are included within the article.
